# Summer aridity rather than management shapes fitness‐related functional traits of the threatened mountain plant *Arnica montana*

**DOI:** 10.1002/ece3.6259

**Published:** 2020-05-08

**Authors:** Nils Stanik, Christian Lampei, Gert Rosenthal

**Affiliations:** ^1^ Department of Landscape and Vegetation Ecology Institute of Landscape Architecture and Environmental Planning University of Kassel Kassel Germany; ^2^ Institute of Landscape Ecology, Biodiversity and Ecosystem Research Group University of Münster Münster Germany

**Keywords:** *Arnica montana*, climate change, fitness‐related performance, gradient analysis, intraspecific variability, plant functional trait, semi-natural mountain grasslands

## Abstract

Semi‐natural mountain grasslands are increasingly exposed to environmental stress under climate change. However, which are the environmental factors that limit plants in these grasslands? Also, is the present management effective against these changes? Fitness‐related functional traits may offer a way to detect changes in performance and provide new insights into their vulnerability to climate change. We investigated changes in performance and variability of functional traits of the mountain grassland target species *Arnica montana* along a climate gradient in Central German low mountain ranges. This gradient represents at its lower end climate conditions that are expected at its upper end under future climate change. We measured vegetative, generative, and physiological traits to account for multiple ways of plant responses to the environment. Using mixed effects and multivariate models, we evaluated changes in trait values among individuals as well as the variability of their populations in order to assess performance under changing summer aridity and different management regimes. Fitness‐related performance of most traits showed strongly positive associations with reduced summer aridity at higher elevations, while only specific leaf area and leaf dry matter content showed no association. This suggests a higher performance level at less arid montane sites and that the physiological traits are less sensitive to this climate change factor. The coefficient of variation of almost all traits declined steadily with decreasing site aridity. We suggest that this reduced variability indicates a lower environmental stress level for *A. montana* toward its environmental optimum at montane elevations, especially because the trait performance increased simultaneously. Surprisingly, management factors and habitat characteristics had only low influence on both trait performance and variability. In summary, summer aridity had a stronger effect to shape the trait performance and variability of *A. montana* under increased environmental stress than management and other habitat characteristics.

## Graphical abstract

We illustrate that summer aridity predicts the performance and variability of fitness‐related functional traits of the threatened mountain grassland species *Arnica montana* under climate change conditions and highlight that increased environmental stress will considerably affect the fitness of *Arnica montana*, while management has only limited influence to mediate impacts.

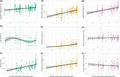

## 1 INTRODUCTION

Anthropogenic climate change is considered a major factor in the current and future biodiversity decline (Butchart et al., 2010). In worst‐case climate change scenarios, mean annual temperature in Europe will increase by 3.7°C (±0.7°C) and summer precipitation will decrease by 1.6% (±5.3%) until the end of the century (Ciscar, Feyen, Ibarreta, & Soria, 2018; IPCC, 2014). Ecosystems that are considered as highly vulnerable to these environmental changes are semi‐natural grasslands in mountain regions (Tasser, Leitinger, & Tappeiner, 2017). For these grasslands, climate‐related changes in species range limits, changes in species composition and in diversity and even local extinctions of species are reported (Gritsch, Dirnboeck, & Dullinger, 2016; Rumpf et al., 2018; Wiens, 2016). As semi‐natural grasslands require management, questions arise whether and to what extent this can influence the maintenance of these ecosystems and species conditions under climate change. Climate change effects interact with management practices because both factors influence and mediate not only the ecosystem functioning but also the resilience of provided ecosystem services (Oliver et al., 2015; Schirpke et al., 2017). Most uncertainties about impacts on species and ecosystems still root in knowledge gaps about the response of species and their fitness‐related functional traits to climatic change (Gillison, 2019). One approach to fill this gap is the space‐for‐time substitution approach that uses the variation of environmental covariates of a species’ natural distribution (space) to learn how functional traits vary with climatic variables that may change in future (time) (Blois, Williams, Fitzpatrick, Jackson, & Ferrier, 2013).

Plant functional traits are an established approach to study responses of species to effects of environmental change such as climate change (Arnold, Kruuk, & Nicotra, 2019; Violle et al., 2007). They are commonly used as proxies for a species’ growth and size with predictive power for its fitness‐related performance in its natural environment (Fraser, Garris, & Carlyle, 2016). For example, many leaf traits reflect aboveground resource acquisition and biomass production (Reich et al., 1999), while generative traits indicate reproductive capacities. Physiological traits (e.g., specific leaf area [SLA] or leaf dry matter content [LDMC]) are strong markers for leaf economics and resource‐use strategies of plants and are considered to be sensitive to changes in climatic conditions (Wright et al., 2004). For instance, studies in subalpine grasslands highlighted the importance of the intraspecific physiological leaf trait variability, which mediated the communities’ functional response to extreme drought (Jung et al., 2014). Therefore, intraspecific variability of functional traits has to be considered when evaluating species climate change responses to buffer negative impacts. This variability is strongly driven by the environmental variation between populations, for example, along environmental gradients (Tautenhahn, Grün‐Wenzel, Jung, Higgins, & Römermann, 2019; Violle et al., 2012).

Functional trait performance, the combined information of functional traits on the vigor of a plant, is closely linked to fitness and usually directly affects fitness components such as survival and reproduction success when performance decreases (i.e., reduced plant height, less inflorescences; Clark et al., 2012; Morris et al., 2008; Silvertown & Charlesworth, 2001). Decreasing functional trait performance in altered environmental conditions would thus signal first steps into a fitness decline of a population. Therefore, fitness‐related functional traits can be used as an early warning system in conservation biology. Furthermore, in addition to the mean, the variability of a trait may also be an indicator for fitness change. High trait variability within a population can indicate a species’ adaptive potential (i.e., increased heritability) when all populations are assessed in the same environment. However, when observed in the natural sites, the environmental variance likely masks heritable variation and high trait variability may rather be an indicator for environmental heterogeneity and less adequate growing conditions (Rowiński & Rogell, 2017; Sinclair & Hoffmann, 2003; Woods, Sgrò, Hercus, & Hoffmann, 1999). Decreasing trait performance of individuals together with increasing phenotypic trait variability among individuals would thus indicate a higher stress level, as both environmental and genotypic variance components increase under stress (Anderson, 2016; Rowiński & Rogell, 2017; Woods et al., 1999).

A representative and endangered plant species of seminatural mountain grasslands is the montane distributed *Arnica montana*, which has experienced a considerable decline during past decades in Europe due to multiple drivers of environmental change (Peppler‐Lisbach & Könitz, 2017; Peppler‐Lisbach & Petersen, 2001). Climate change is considered to have an increased impact on the species as habitat fragmentation and the limited elevation of low mountain ranges in Central Europe do not allow the species to migrate upslope to compensate less suitable future conditions at their current distribution (Pauli et al., 2012). In addition, it is reported that slug herbivory acts as a limiting factor for *Arnica*'s geographical range toward lowland sites by causing considerable leaf damages (Bruelheide & Scheidel, 1999). Overall threats for *A. montana* across elevations are isolation and small‐sized populations with low genetic diversity (Duwe, Muller, Borsch, & Ismail, 2017), because the reproductive and genetic fitness of *Arnica* populations are strongly influenced by population size and its demographic structure (Kahmen & Poschlod, 2000; Maurice, Colling, Muller, & Matthies, 2012; Maurice, Matthies, Muller, & Colling, 2016). However, elevation and linked climatic factors as well as management are considered as equally important factors that influence the vegetative and generative performance of *A. montana* individuals because environmental conditions and biotic interactions are expected to become more suitable at higher elevations (Mardari et al., 2019). Further positive contributions to *Arnica*'s population demography can also be expected from late low‐intensive management, as frequently suggested in agri‐environment measures for their kinds of habitats (Schwabe, 1990; Schwabe et al., 2019). The trait performance and variability of individuals and populations, respectively, are therefore key measures to determine the species’ future response and are vital to evaluate its vulnerability to climate change.

The aim of this study was to detect and quantify how functional traits of the threatened mountain plant *A. montana* change along an elevation‐based climate gradient in low mountain ranges of Central Europe. Here, temperatures increase and summer precipitation decreases markedly with lower elevation resulting in a strong aridity difference. This summer aridity gradient is therefore well suited to study the potential future plant performance of *A. montana* under climate change conditions by using a space‐for‐time approach. Furthermore, the interaction of climate factors with the management of the sites is of high importance because they also shape habitat qualities. With this approach, we examined effects of climate conditions along the gradient from lowland to montane *A. montana* populations in their Central European outer alpine distribution area. The investigated climate gradient represents at its lower end climate conditions of Central Europe that are expected at its upper end in the future (Ciscar et al., 2018). We therefore focus on the question: Do the trait performance of maternal *A. montana* individuals and the trait variability of their populations change along this climate gradient? We predict that (a) the performance of all tested traits increases toward higher viability as summer aridity decreases with higher elevation. The more favorable, humid mountain climate at high elevations should provide better growing conditions for this predominantly montane distributed plant species. We further predict that (b) the populations’ variability of traits decreases at the same gradient with decreasing summer aridity. Traits should perform more uniform toward higher elevations in the mountain range as the summer climate becomes less stressful for mountain plants. Furthermore, we expect that (c) suitable management as well as habitat and site characteristics contribute additionally to the performance and level the variability of traits by promoting favorable habitat qualities. These analyses will shed light on the specific ecological response of an endangered species that is particularly exposed to climate change and will indicate potential demands for future conservation.

## 2 MATERIALS AND METHODS

*Arnica montana* L. (Asteraceae) is distributed across Europe and grows in acidic oligotrophic grasslands, mainly *Nardetalia strictae* communities (Peppler‐Lisbach & Petersen, 2001), and heathlands in colline to montane regions (Heijne, Hofstra, Heil, van Dam, & Bobbink, 1992; Meusel, Jäger, & Weinert, 1992). The sporophytic self‐incompatible herbaceous perennial forms rhizomes with summer‐green rosettes for its vegetative reproduction and produces wind‐dispersed seeds (achenes) (Luijten, Kéry, Oostermeijer, & den Nijs, 2002). *Arnica montana* is an important medicinal plant and protected by the EU Habitats Directive (Annex V) due to its ongoing decline (European Council, 1992). Therefore, broad conservation efforts are currently undertaken across Europe to protect its wild populations.

We surveyed populations of *A. montana* (*n* = 52) in *Nardetalia* grasslands along an elevation gradient from 281 m to 929 m a.s.l. in Central Germany (Southeast Hesse and Northern Bavaria), each of them of approximately the same size (Supporting information S1). We selected populations in the study area using the random sampling tool in QGIS 3.4 (QGIS Development Team, 2019) and started the sampling from south to north and from the lowest to the highest elevated sites to minimise sampling bias. Based on the multi‐annual monthly mean precipitation and multi‐annual monthly mean air temperature, we characterised the surveyed elevation gradient climatically with data of the reference period 1981–2010 provided by the DWD Climate Data Center (2019a, 2019b). The covered temperature and precipitation range of the gradient are 5.6–9.2°C and 707 to 1,320 mm, respectively. Based on these data, we calculated the population sites’ mean aridity index by Martonne (1926) for the summer period (April–September), in which higher scores indicate less arid conditions. For summer‐green plants like *A. montana*, the summer period is of particular importance because their whole vegetative and generative development cycle takes place within this period (Kahmen & Poschlod, 1998).

Within the surveyed *A. montana* populations, we selected randomly 12 adult rosettes (individual level), separated by a distance of at least one meter, and measured vegetative traits (vegetative height, leaf number, leaf length, leaf width, leaf area) and generative traits (flower stem height, number of inflorescences, and diameter of the uppermost inflorescence). From these 12 individuals, we selected six to determine leaf dry biomass, SLA, and LDMC as physiological traits (cf. Ley et al., 2018). In general, the whole procedure of measurement, sampling, and further processing was performed according to the standard protocol of Pérez‐Harguindeguy et al. (2013). Leaf length, leaf width, leaf area, and leaf biomass were measured at the biggest leaf of the rosette; leaf area was determined with the program ImageJ2 (Rueden et al., 2017).

At each population site, we recorded habitat characteristics (herb layer cover and height, moss cover and height, litter cover and height, cover of grasses and of bare soil, and topsoil pH value) in a 2.5 m × 2.5 m plot around the *A. montana* stands. Mixed soil samples of the upper 0–10 cm (auger diameter 5 cm) were collected and thoroughly mixed to ensure homogeneity for the electrometrical measurement of topsoil pH in deionised water and 1 N KCl solution.

In the study region, elevation change results in strong changes in temperature, precipitation, and summer aridity. Elevation was negatively correlated with air temperature (*r*_s_ = −.98, *p* < .001) and positively with precipitation (*r*_s_ = .82, *p* < .001) and the calculated index of summer aridity (*r*_s_ = .95, *p* < .001). High summer aridity index scores at higher elevations represent, therefore, a decreased summer aridity at these sites (Martonne, 1926). Differences in air temperature between the warmest and coldest sites amount to 3.6°C, whereas precipitation changes amount to a margin of 613 mm between sites that receive the lowest and the highest amount of precipitation. The difference in summer aridity is 2.2 mm/°C along the considered elevation gradient. Since summer aridity is highly associated with the change of elevation, temperature, and precipitation, this factor is used as an appropriate proxy for the climatic water balance to evaluate effects of changing climate conditions on *A. montana* (for model results and estimates based on elevation, see Supporting information S4).

All statistical analyses were conducted in r 3.6.1 (R Core Team, 2019). Data exploration was carried out following the protocol described in Zuur, Ieno, and Elphick (2010). Leaf length and leaf width were excluded prior to the analysis as these were strongly correlated with leaf area (*r*_p_ = .82 and *r*_p_ = .81, *p* < .001, respectively). Vegetative height and leaf biomass were log + 1‐transformed to obtain normal distribution. We analysed each trait independently to evaluate its relationship with elevation‐related summer aridity and management. Depending on the measured response trait, we fitted at individual level mixed effects models (linear mixed effects models for vegetative height, leaf area, flower stem height and inflorescence diameter, leaf biomass, SLA, and LDMC; generalised mixed effects models with Poisson distribution for inflorescence number; orthogonal polynomial mixed effects model for leaf number). Each model contained the trait as dependent variable and summer aridity (mm/°C, mean centered and scaled), management type (factor with two levels, grazing or mowing), and management time (factor with three levels) as independent variable and the population ID as random intercept. Management time was defined by the management date of a year according to agri‐environment scheme regulations of the grassland sites (early: before 15 June, intermediate: 15 June to 15 July, and late: after 15 July). Model selection was based on Akaike's information criterion (AIC), where ∆15 AIC was set as minimum for a significant model improvement (c.f. Harrison et al., 2018). Mixed effects models were fitted using restricted maximum likelihood with the package “lme4” (Bates, Mächler, Bolker, & Walker, 2015).

To assess the intraspecific variability of traits among populations (population level), we calculated the coefficient of variation (CV) for each trait and fitted it as response variable in linear models with summer aridity, management type, and management time as predictor variables. Due to the nonlinear relationship of CV for vegetative height, an orthogonal polynomial model was used. We analysed changes in habitat characteristics along the gradient by using multivariate linear models with elevation, management time, and management type as independent variables, for which we log + 1‐transformed herb layer height, grass cover, moss cover, litter cover and height as well as bare soil to improve normality for these habitat parameters. We tested for potential interactions between independent variables, which did not yield a significant improvement of the models based on the set ∆AIC threshold. Therefore, we did not include interactions in the final models. To evaluate which site and habitat characteristics are best predictors across traits of the *A. montana* populations, we applied a multivariate analysis of variance (PERMANOVA with 999 permutations; function: adonis, package “vegan,” Oksanen et al., 2019) based on the Bray–Curtis dissimilarity measure for population's trait averages and CV values.

## 3 RESULTS

With the space‐for‐time substitution approach along an elevation‐based climate gradient of ∆648 m, we evaluated the response and variability of fitness‐related plant functional traits of *A. montana* to changing climate conditions. Fitted models revealed significant response pattern across traits at individual level and their variability at population level along the summer aridity gradient. Management factors and habitat characteristics were evenly distributed in the covered range but showed to have only low influence on trait performance and variability, respectively.

### 3.1 Influences of summer aridity and management on functional trait performance at individual level

**Figure 1:**
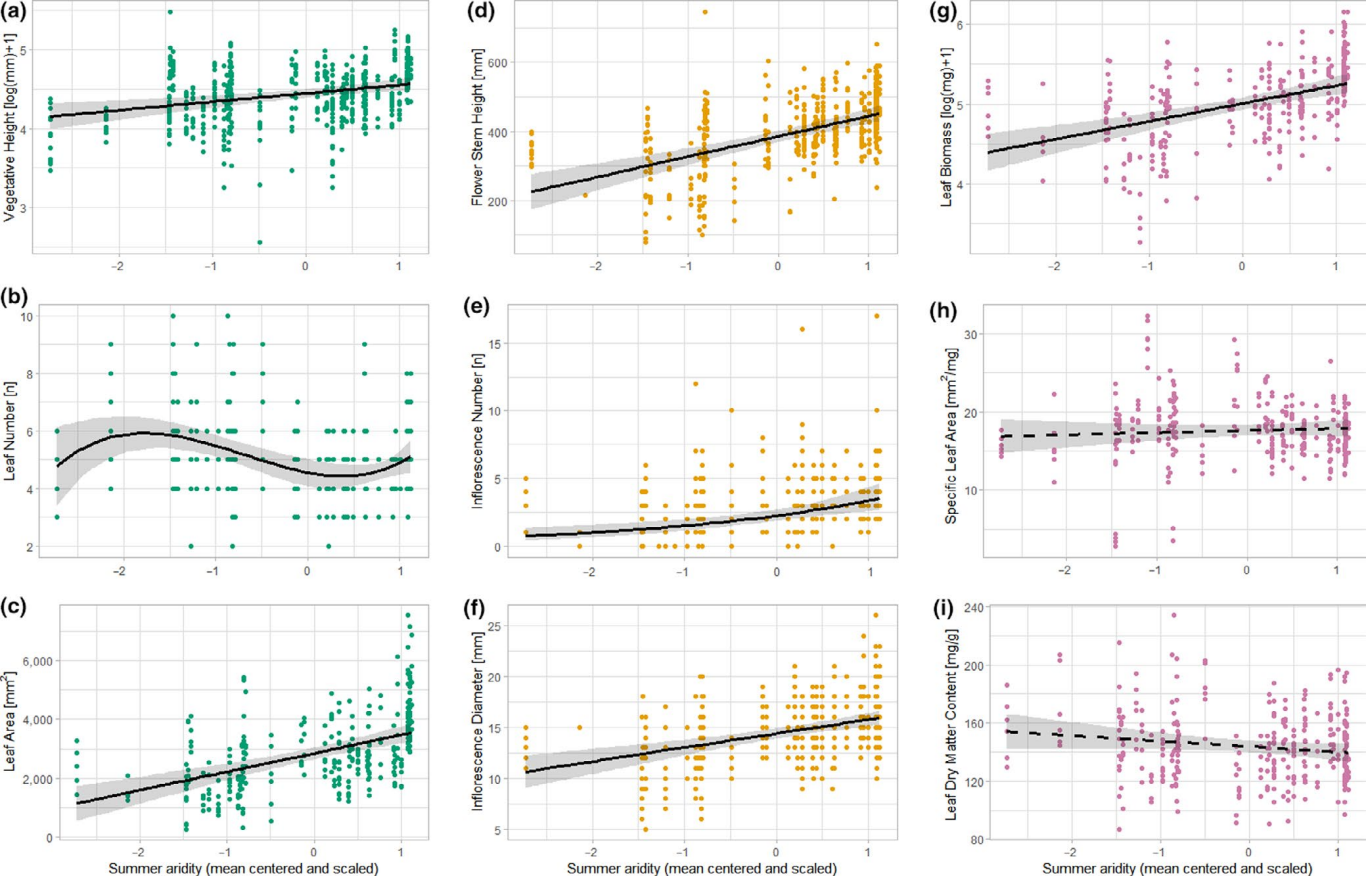
Changes in the plant functional trait performance of *Arnica montana* with decreasing summer aridity at higher elevated montane sites (lower summer aridity is indicated by higher scores). Solid lines indicate significant model trends (*p* < .05) and dashed lines nonsignificant trends with the gray‐shaded 95% confidence interval. Figure parts (a–c) show trends for vegetative traits, (d–f) for generative traits, and (g–i) for physiological traits. See Supporting information S3 for detailed model results

Along the climate gradient, traits of all trait groups showed significant associations with environmental variables of which summer aridity had the highest predictive power (Figure 1, Supporting information S3). Only SLA (*β* = 0.27, *p* = .474) and LDMC (*β* = −3.74, *p* = .086) were not significantly influenced by summer aridity. Vegetative height, leaf area, flower stem height, inflorescence number, and inflorescence diameter as well as leaf biomass increased with decreasing summer aridity. Leaf number showed a nonlinear response with a decrease from low‐to‐intermediate aridity scores (*β* = −6.98, *p* = .017) and strong increase at high aridity scores (*β* = 8.75, *p* = .006). In contrast, management type and management time played minor roles for the performance of traits. Only SLA showed a negative association to management type. It was significantly lower in plants that were affected by mowing than those that were exposed to grazing (*β* = −3.05, *p* = .006). Hence, the decrease of summer aridity with increasing elevation could be highlighted as an important climate‐related predictor for the trait performance of *A. montana*.

### 3.2 Influences of summer aridity and management on functional trait variability at population level

**Figure 2:**
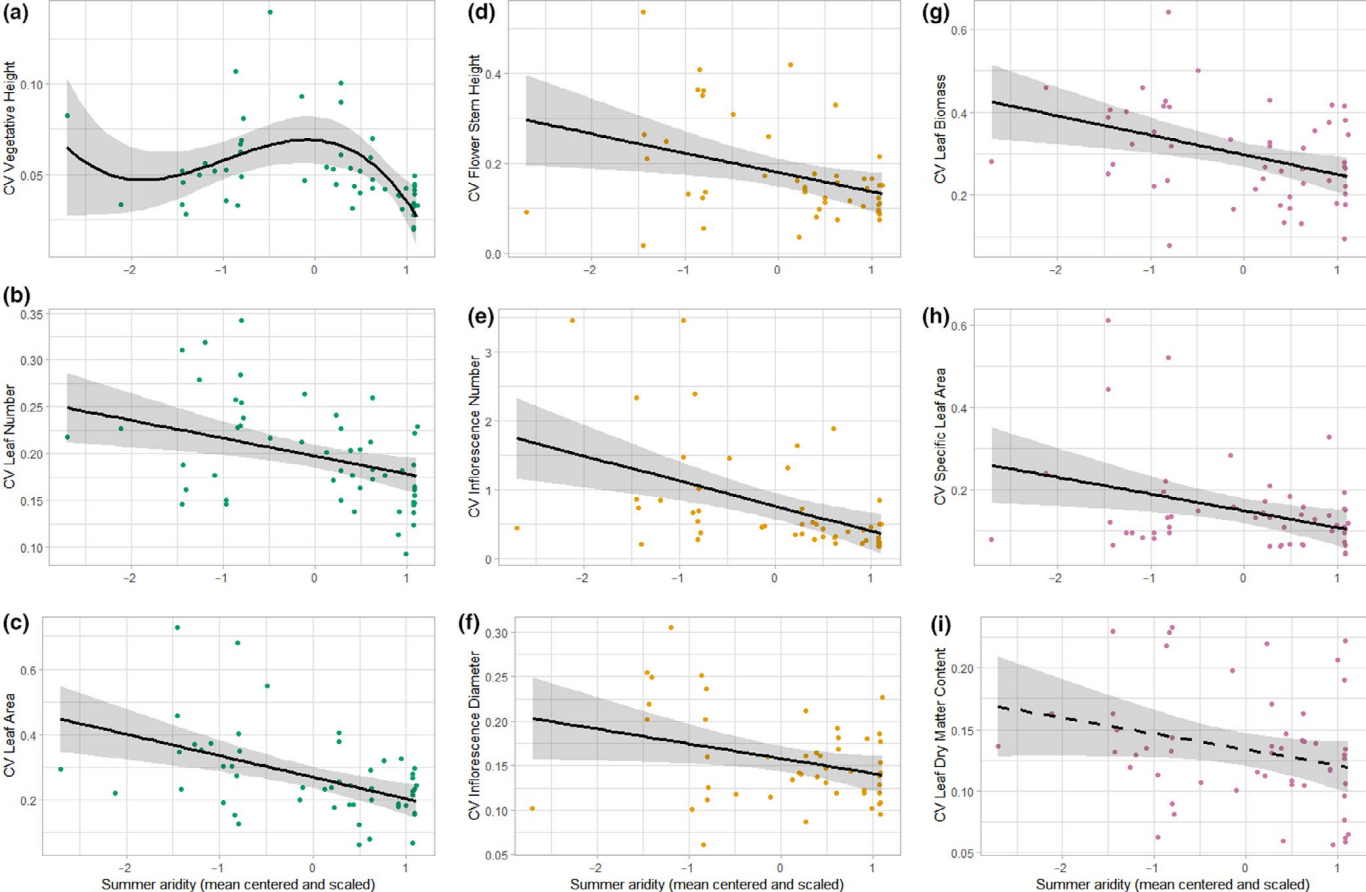
Changes in the variability (coefficient of variation, CV) of plant functional traits of *Arnica montana* with decreasing summer aridity at higher elevated montane sites (lower summer aridity is indicated by higher scores). Solid lines indicate significant model trends (*p* < .05) and dashed lines nonsignificant trends with the gray‐shaded 95% confidence interval. Figure parts (a–c) show trends for vegetative traits, (d–f) for generative traits, and (g–i) for physiological traits. See Supporting information S3 for detailed model results

Next, we tested whether the populations CV of traits changed with summer aridity. Among vegetative traits, leaf area showed the highest, while vegetative height showed the lowest variability. In the generative trait group, inflorescence number was the most variable and inflorescence diameter the least variable trait. Among physiological traits, leaf biomass showed the highest and LDMC the lowest variability. The variability of most traits decreased consistently with decreasing summer aridity at higher elevations, while only LDMC showed a nonsignificant decrease (*β* = −0.01, *p* = .073; Figure 2, Supporting information S3). The strongest variability decrease with lower summer aridity showed the trait inflorescence number (*β* = −0.37, *p* < .001). In contrast to these consistent linear response patterns, the variability of vegetative height showed a nonlinear response. When looking at management factors, only management time influenced the variability of leaf number: variability at sites with late management was lower than at early managed sites (*β* = −0.07, *p* < .05). All other traits were influenced neither by management type nor by management time. Hence, the decrease in variability of traits among populations can be primarily attributed to the change of summer aridity along the climate gradient.

### 3.3 Overall trait values in relation to site and habitat characteristics

Of the nonclimate‐related characteristics, only herb layer height showed a decrease along the gradient (*β* = −0.00029, *p* < .05; i.e., ∆4.37 cm). Furthermore, there were no differences in habitat characteristics between management types and management times (Supporting information S2). With the multivariate PERMANOVA test on the influence of site and habitat characteristics on the population trait average and CV, we revealed an additional set of shaping factors (Table 1). Average trait values were additionally influenced by site characteristics, like geological substrate, bare soil, and topsoil pH, and management time. In contrast, the variability of traits was additionally influenced by the habitat characteristics moss height and bare soil. Elevation was highlighted as the most contributing factor that shapes both populations’ average and CV trait values of *A. montana*, while summer aridity, which had been used in prior analyses, was eventually deleted during backwards elimination due to its strong collinearity to elevation. Being a multifactor environmental variable, elevation correlated with the trait averages and CV slightly better than summer aridity, however, with less predictive power for generalising these results.

**Table 1:** Influences of site and habitat on population's mean trait values (a) and population's variability of trait values (b) based on Bray–Curtis dissimilarity

Site and habitat characteristics	*df*	*F*	*R* ^2^	*p*
(a)				
Elevation	1	75.003	.48	**<.001**
Geological substrate	1	19.585	.12	**<.001**
Management time	2	4.510	.06	**.013**
Bare soil	1	5.215	.03	**.019**
Topsoil pH (H_2_O)	1	3.564	.02	.053
Residuals	45		.29	
(b)				
Elevation	1	17.3141	.23	**<.001**
Moss height	1	4.0710	.05	**.014**
Management time	2	2.3321	.06	.057
Bare soil	1	2.7693	.04	**.045**
Residuals	46		.61	

Significant influences are set in bold (*p* < .05).

## 4 DISCUSSION

The results of this study emphasise the importance of summer aridity for both the trait performance of individuals and the populations’ trait variability of the endangered mountain plant species *A. montana*. Almost every tested vegetative, generative, and physiological trait responded positively in its performance to a better suiting climatic water balance during summer at higher elevations, while only SLA and LDMC showed no association to this climate factor. This supports our first prediction that the performance of traits increases towards higher viability as summer aridity decreases with higher elevation. The better suited growing conditions in the less arid montane climate allow this mountain grassland species to develop a higher performance level in the form of higher‐growing individuals with larger leaves, bigger flowers, and higher leaf biomass. Simultaneous to the increased viability, the populations’ trait variability of almost all traits (except LDMC) declined with reduced summer aridity at higher elevated sites. The lower variability of traits at higher elevations supports our second prediction by indicating a lower environmental stress level for *A. montana* in montane regions (Sinclair & Hoffmann, 2003; Woods et al., 1999). In contrast, management and habitat characteristics had only low influence on fitness‐related traits of the species. In this study, to our surprise, we did not detect the expected mitigating effects of management or of other advantageous habitat characteristics that are capable to reduce climate stress for the species.

Summer aridity has been identified to play a major role in the ecological response pattern of fitness‐related plant functional traits of the summer‐green *A. montana* to a changing climate. Aridity receives increasingly attention in climate change research as an important parameter of the water regime because it integrates both temperature and precipitation, which are generally considered as single drivers of plant functional traits (Moles et al., 2014). An increased aridity‐driven water stress level during summer severely hinders plant development and affects plant regeneration after disturbance events like management actions (Puig‐Gironès, Brotons, & Pons, 2017). Therefore, especially for Europe with its heterogeneous elevation pattern and east–west continentality gradient, plant–aridity relations generate more generalisable and transferable findings of climate change impacts than using elevation or other elevation‐related climate factors to predict plant responses (Körner, 2007).

The trait performance change of *A. montana* individuals along the tested aridity gradient indicates higher environmental stress due to an increased water stress level at sites with higher summer aridity. This suggests that the *A. montana* sites in the German lower mountain ranges rank at the dry end of the species ecological niche. However, similarly stress‐induced declines in vital rates of *A. montana* populations were also reported at the species’ other distribution range edges as response to environmental stress. For example at the northern distribution range edge, which is characterised by high humidity and moisture, population growth rates and flowering decreased with increasing precipitation and increasing temperatures (Vikane, Rydgren, Jongejans, & Vandvik, 2019). Likewise in another study, the flowering performance (i.e., No. of inflorescences per flowering rosette) of populations decreased at its cold upper altitudinal range limit above 2,000 m a.s.l. and resulted in a shift toward vegetative reproduction (Mardari et al., 2019). These examples, though from the opposite end of the species ecological niche, support our results at warmer and dryer, hence more arid sites, that *A. montana* responds very sensitive to increased stress levels under suboptimal habitat conditions at the edges of their natural distribution by reducing life performances.

However, not all traits are similarly sensitive to environmental stress. In the current study, no significant responses were observed for SLA and LDMC, which suggest that leaf economic traits of *A. montana* are less sensitive to increased aridity. This response is congruent with the intraspecific trait–temperature relationship of other mountain plant species (Rosbakh, Römermann, & Poschlod, 2015). However, results from a greenhouse experiment with *A. montana* suggest that LDMC responds not until a higher drought level, which is not reached in the present study (N. Stanik et al., unpublished data). Our study sites show moreover no considerable soil fertility gradient as topsoil pH and most nutrient‐sensitive habitat characteristics were constant across sites (Stevens et al., 2011), which thus contributes to the identified stability of growth rate indicators (Ordoñez et al., 2009). Hence, these findings underpin the major influence of climate at sites with homogeneous habitat conditions on the morphometrical performance of *A. montana* towards its lower elevated distribution range.

In the German lower mountain range, the summer period at higher elevations is characteried by lower temperature variation and more ambient soil moisture and humid conditions than lower elevated sites, hence a more constant climate (Beniston, 2006). These less variable climate conditions contribute to low intraspecific variability of traits in a species’ environmental optimum (Albert et al., 2010). Accordingly, the lowland populations of *A. montana* with higher climate variability (e.g., with higher temperatures and a heterogeneous precipitation regime) showed a higher variability of traits than upland populations, triggered by phenotypic adjustments of these traits (Rowiński & Rogell, 2017⁠⁠; Woods et al., 1999). This stresses their hereby revealed adaptive capacity to buffer negative impacts of climate change (Luo et al., 2019; Wellstein et al., 2013). Nonetheless, this illustrates that lower climate variability contributes to a higher performance and an even trait variability of mountain plants like *A. montana* towards their distribution optimum.

The weak influence of management and habitat characteristics in our study to *Arnica*'s fitness contrasts with their previously identified effects. Many studies that highlight the importance of management included diverse management intensities, which ranged from more intensively managed to fallow sites, in which *A. montana* has either bad or advantageous growing conditions (e.g., Kahmen & Poschlod, 1998⁠; Maurice et al., 2012). Instead, we incorporated only management categories according to agri‐environmental scheme regulations, which are considered to be beneficial for the species, for example, by favoring the plants’ seed maturation, and effective in its habitat conservation (Schwabe et al., 2019). Despite the different management regimes, management intensity at the surveyed sites appears to be quite homogeneous resulting in minor differences of habitat characteristics that did not trigger growth form differences of *A. montana* as investigated by Schwabe (1990). In our study, management time was the only factor that influenced the performance and variability of the considered set of traits. Late management provides generally more time for the overall vegetative and generative development and resource acquisition of perennial clonally growing species (Liu, Liu, & Dong, 2016). However, our findings show that management, which currently maintains suitable habitats for *A. montana*, will have limited power to promote the performance of single traits and the overall trait performance, when environmental conditions become less suitable under future climate change.

Limitations of the space‐for‐time approach to study climate change impacts on the fitness of plants may arise by insufficient detection and selection of samples (Roth, Allan, Pearman, & Amrhein, 2018), as well as by shifts in ontogeny and phenology along the gradient when collecting data. For instance, differences in the ontogenetical and phenological state of individuals between populations can influence the variability of traits (Römermann, Bucher, Hahn, & Bernhardt‐Römermann, 2016). Here, we tried to minimise this influence by organising the whole sampling in a concise period, in which the individuals were fully developed, and by arranging the sampling from south to north and from the lowest (i.e., the earliest in development) to the highest elevated sites. The consideration of the calendar week of the collected data as a random factor to account for this potential bias did not improve the models fit, which suggests only a minor influence of phenology. The investigated climate gradient of this study is based on elevation at which not only aridity but also several other climate parameters change, which might cause changes in plants fitness‐related traits (Körner, 2007). However, we attribute changes among populations to summer aridity as a water stress indicator, which is highly correlated with elevation. Moreover, we assumed that environmental changes along the climate gradient are stronger than potential genetic differences between populations and thereof developed adaptations (Maurice et al., 2016). The identified changes might have been therefore even more pronounced than estimated when considering these potential adaptations in the interpretation. Finally, in contrast to previous findings we did not observe any leaf damages from herbivory, which could otherwise have influenced the trait performance at lowland sites. Due to the relatively short sampling period, age‐ and season‐specific herbivory damage may be not observed in our study (Scheidel & Bruelheide, 2004).

## 5 CONCLUSION

Our findings demonstrate the high importance of summer aridity along an elevation gradient to predict the performance and variability change of fitness‐related traits of a threatened mountain plant species by climate change. Contrarily, management and habitat characteristics had only limited influence to mediate environmental change impacts on most traits. However, even if the considered management actions may not promote direct support against major climatic changes, they are required to maintain the habitat of semi‐natural grasslands at many sites. The identified intraspecific trait–climate relationships indicate the present potential of *A. montana* to adapt plastically to climate change. The here investigated response pattern and associated climate drivers behind these relationships are relevant for a more realistic modeling of distributions of endangered species (Parolo, Rossi, & Ferrarini, 2008). Furthermore, the findings renew issues about the relative importance of small‐scale abiotic environmental and microclimatic factors as agents to either foster or hamper species fitness with declining site suitability under future climate change (Sebastiá, 2004). Consequently, nature conservation should consider the limited scope of management in the species future protection and should increasingly consider fitness‐related impacts by climate change on vulnerable species in management planning, identify high impact and refugial areas, and expand efforts to stabilise climatically exposed populations.

## AUTHOR CONTRIBUTION

**Nils Stanik:** Conceptualization (lead); Formal analysis (lead); Methodology (lead); Writing‐original draft (lead); Writing‐review & editing (lead). **Christian Lampei:** Formal analysis (supporting); Methodology (supporting); Supervision (supporting); Writing‐original draft (supporting); Writing‐review & editing (supporting). **Gert Rosenthal:** Conceptualization (supporting); Formal analysis (supporting); Methodology (supporting); Project administration (lead); Supervision (lead); Writing‐original draft (supporting); Writing‐review & editing (supporting).

## Supporting information

Supplementary Material

## Data Availability

The plant functional trait data of *A. montana* (individuals and populations) and data about the populations site and habitat characteristics are published at Zenodo.org with https://doi.org/10.5281/zenodo.3701492.
