# Supplementary material for: Summer aridity rather than management shapes fitness‐related functional traits of the threatened mountain plant Arnica montana
Source: Ecol Evol. 2020 May 8;10(11):5069–78. doi: 10.1002/ece3.6259 (PMC7297756; doi:10.1002/ece3.6259)
Supplement: Supplementary file 1 — Supplementary Material [file ECE3-10-5069-s001.docx]

# **Supporting information S1**: Information about site characteristics of the considered *Arnica montana* populations (table and location map).

### Figure 1-S1. Location map of considered *Arnica montana* populations with aridity-coded legend


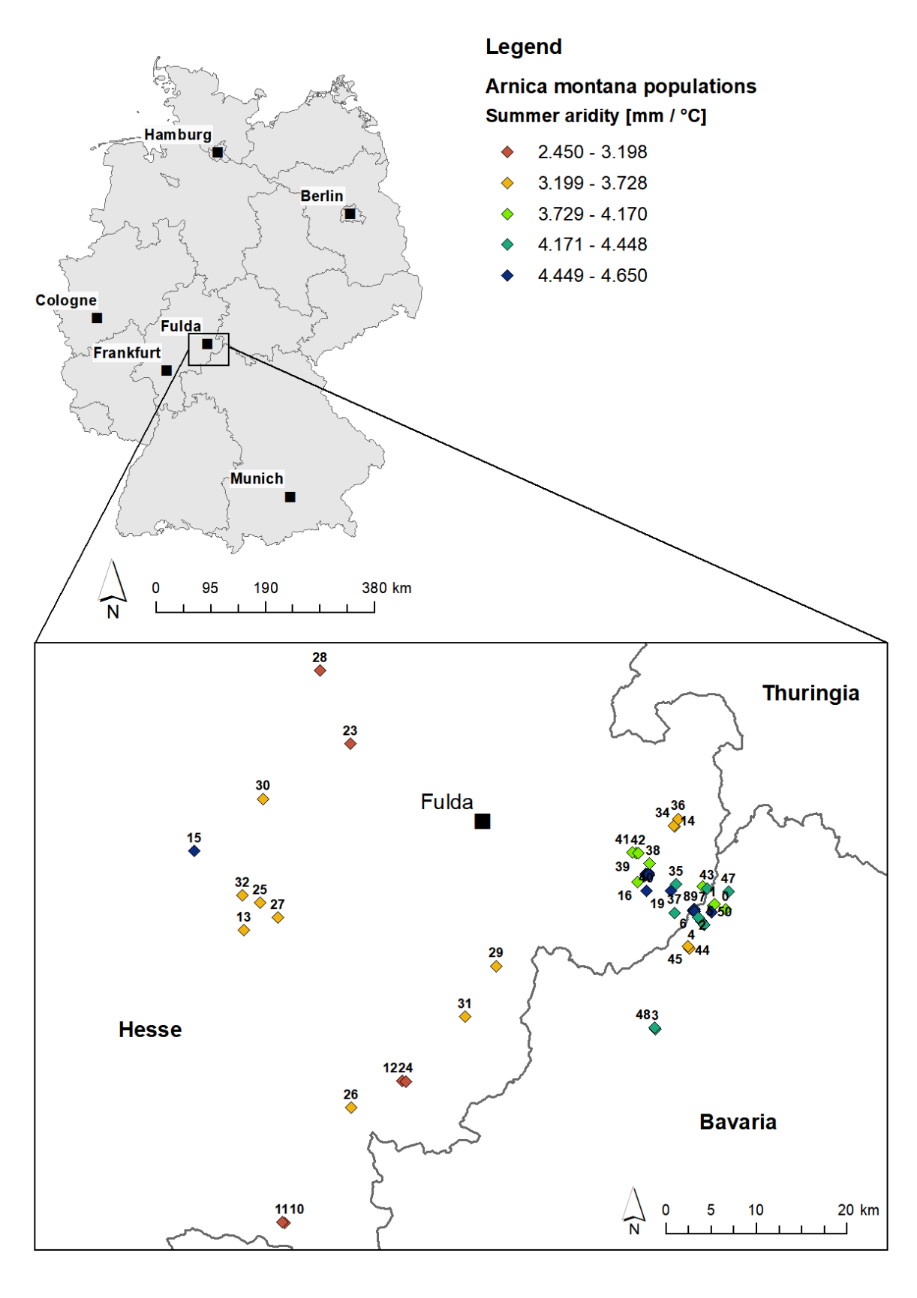


### Figure 2-S1 Location map of considered *Arnica montana* populations with elevation-coded legend


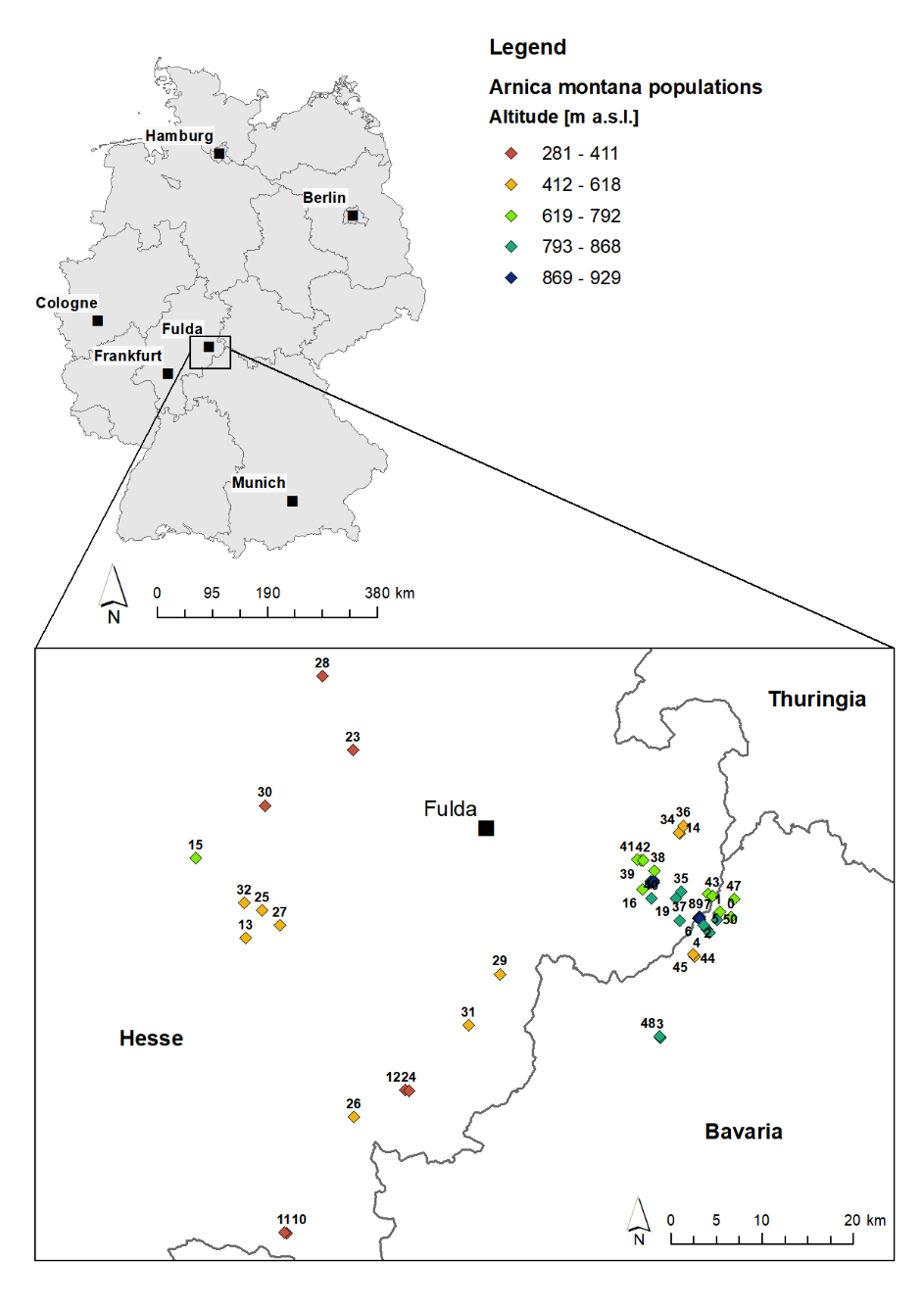


### Table 1-S1. Location details of the considered *Arnica montana* populations

| **Population ID** | **Map ID** | **Elevation** [m a.s.l] | **Elevation** [m a.s.l] (mean centered and scaled) | **Management  type** | **Management  time** | **Multi-annual mean air temperature** [°C] [(DWD Climate Data Center, 2019b)](#_CTVL001d69e3e64f2e54092b8225af4bea5b1db) | **Multi-annual mean precipitation** [mm] [(DWD Climate Data Center, 2019a)](#_CTVL0018c64424451be44309519556e6e16b5b1) | **Summer aridity** [mm/°C] | **Summer aridity** [mm/°C]  (mean centered and scaled) |
| --- | --- | --- | --- | --- | --- | --- | --- | --- | --- |
| BY500.1 | 44 | 593 | -0.48976618 | mowing | intermediate | 7.4 | 1040 | 3.453399109 | -0.964751719 |
| BY600.1 | 45 | 600 | -0.45532358 | mowing | intermediate | 7.4 | 1040 | 3.453399109 | -0.964751719 |
| BY700.3 | 0 | 776 | 0.41066175 | grazing | intermediate | 6.2 | 1155 | 4.082379515 | 0.126360717 |
| BY700.6 | 1 | 792 | 0.48938769 | mowing | intermediate | 6.0 | 1220 | 4.367989072 | 0.621816753 |
| BY700.7 | 46 | 787 | 0.46478584 | mowing | late | 6.2 | 1164 | 4.125980842 | 0.201997334 |
| BY700.8 | 47 | 790 | 0.47954695 | mowing | intermediate | 6.0 | 1177 | 4.233074533 | 0.387776186 |
| BY800.2 | 48 | 820 | 0.79445071 | mowing | intermediate | 6.2 | 1196 | 4.294676338 | 0.494638807 |
| BY800.3 | 3 | 828 | 0.98142481 | mowing | intermediate | 6.2 | 1196 | 4.294676338 | 0.494638807 |
| BY800.6 | 4 | 835 | 0.62715809 | mowing | intermediate | 5.9 | 1229 | 4.372647138 | 0.629897249 |
| BY800.8 | 5 | 850 | 0.66652106 | mowing | intermediate | 6.1 | 1195 | 4.242040698 | 0.403330079 |
| BY800.9 | 50 | 859 | 0.70096366 | mowing | late | 5.9 | 1229 | 4.372647138 | 0.629897249 |
| BY800.10 | 21 | 854 | 0.77476922 | mowing | late | 5.7 | 1263 | 4.554041924 | 0.944568588 |
| BY800.12 | 2 | 892 | 0.81905256 | mowing | late | 5.7 | 1276 | 4.628094977 | 1.073030788 |
| BY900.1 | 6 | 900 | 1.02078778 | mowing | late | 5.7 | 1276 | 4.628094977 | 1.073030788 |
| BY900.2 | 7 | 904 | 1.04046927 | mowing | late | 5.7 | 1276 | 4.628094977 | 1.073030788 |
| BY900.3 | 49 | 911 | 1.07491187 | mowing | late | 5.7 | 1276 | 4.628094977 | 1.073030788 |
| BY900.4 | 8 | 913 | 1.08475261 | mowing | late | 5.7 | 1276 | 4.628094977 | 1.073030788 |
| BY900.5 | 9 | 922 | 1.12903595 | mowing | late | 5.6 | 1286 | 4.65047456 | 1.111853367 |
| HE200.1 | 23 | 281 | -2.024922 | mowing | late | 8.8 | 707 | 2.450227002 | -2.704986469 |
| HE200.2 | 10 | 284 | -2.01016089 | mowing | late | 9.2 | 1004 | 3.172751213 | -1.451600603 |
| HE200.3 | 11 | 292 | -1.97079792 | mowing | late | 9.2 | 1004 | 3.172751213 | -1.451600603 |
| HE300.1 | 28 | 324 | -1.81334604 | mowing | intermediate | 8.2 | 766 | 2.786857985 | -2.121021932 |
| HE300.2 | 24 | 360 | -1.63621268 | mowing | late | 8.3 | 968 | 3.181808099 | -1.435889334 |
| HE300.3 | 12 | 364 | -1.61653119 | mowing | late | 8.3 | 981 | 3.19837527 | -1.407149732 |
| HE400.1 | 30 | 411 | -1.38527375 | grazing | early | 7.9 | 1057 | 3.280767272 | -1.264221689 |
| HE400.2 | 25 | 438 | -1.25242372 | mowing | intermediate | 7.9 | 1136 | 3.524983288 | -0.840572353 |
| HE400.3 | 29 | 445 | -1.21798112 | mowing | early | 7.7 | 942 | 3.317953114 | -1.199714219 |
| HE400.4 | 13 | 457 | -1.15893667 | grazing | intermediate | 8.0 | 1101 | 3.538415379 | -0.817271275 |
| HE400.5 | 27 | 464 | -1.12449407 | mowing | intermediate | 7.8 | 1109 | 3.510099163 | -0.866392321 |
| HE400.7 | 26 | 472 | -1.0851311 | mowing | late | 7.9 | 1072 | 3.542498276 | -0.810188542 |
| HE500.1 | 31 | 504 | -0.92767922 | grazing | intermediate | 7.6 | 995 | 3.38097256 | -1.090392371 |
| HE500.2 | 32 | 506 | -0.91783848 | mowing | intermediate | 7.7 | 1159 | 3.728173046 | -0.488092579 |
| HE500.3 | 34 | 577 | -0.56849212 | grazing | intermediate | 7.3 | 982 | 3.547261137 | -0.801926256 |
| HE500.4 | 14 | 560 | -0.65213843 | grazing | early | 7.3 | 982 | 3.547261137 | -0.801926256 |
| HE600.1 | 36 | 618 | -0.3667569 | mowing | intermediate | 7.1 | 985 | 3.557549257 | -0.784079125 |
| HE600.2 | 15 | 698 | 0.0268728 | mowing | late | 6.5 | 1320 | 4.583629903 | 0.995895802 |
| HE700.2 | 41 | 744 | 0.25320987 | mowing | intermediate | 6.5 | 1127 | 4.170367868 | 0.278996927 |
| HE700.4 | 16 | 767 | 0.36637841 | grazing | early | 6.7 | 1077 | 3.945495223 | -0.111096844 |
| HE700.6 | 33 | 743 | 0.2482895 | grazing | intermediate | 6.7 | 1116 | 3.926624001 | -0.143833357 |
| HE700.7 | 42 | 767 | 0.36637841 | mowing | intermediate | 6.5 | 1127 | 4.170367868 | 0.278996927 |
| HE700.9 | 38 | 770 | 0.38113953 | grazing | intermediate | 6.5 | 1094 | 4.139632138 | 0.225678672 |
| HE700.10 | 39 | 771 | 0.3860599 | mowing | intermediate | 6.5 | 1127 | 4.170367868 | 0.278996927 |
| HE700.12 | 43 | 787 | 0.46478584 | grazing | intermediate | 6.0 | 1208 | 4.361010391 | 0.609710612 |
| HE800.1 | 37 | 803 | 0.54351178 | mowing | intermediate | 6.3 | 1198 | 4.258386878 | 0.43168632 |
| HE800.2 | 35 | 818 | 0.61731734 | mowing | intermediate | 6.2 | 1228 | 4.448584935 | 0.761628975 |
| HE800.5 | 19 | 820 | 0.62715809 | mowing | late | 6.0 | 1243 | 4.533909478 | 0.909644189 |
| HE800.7 | 40 | 828 | 0.66652106 | mowing | intermediate | 6.0 | 1215 | 4.536797586 | 0.914654283 |
| HE800.11 | 17 | 868 | 0.86333591 | mowing | late | 5.7 | 1189 | 4.634837996 | 1.084728119 |
| HE800.12 | 18 | 882 | 0.9322211 | mowing | late | 5.7 | 1189 | 4.634837996 | 1.084728119 |
| HE900.3 | 20 | 916 | 1.09951372 | mowing | late | 5.7 | 1189 | 4.634837996 | 1.084728119 |
| HE900.4 | 21 | 918 | 1.10935447 | mowing | late | 5.7 | 1189 | 4.634837996 | 1.084728119 |
| HE900.5 | 22 | 929 | 1.16347855 | mowing | late | 5.7 | 1189 | 4.634837996 | 1.084728119 |

Note: For definition of management times, see materials and methods section of the paper.

# **Supporting information S2**: Tables with results of linear models for changes in habitat/site characteristics with elevation, management type and management times.

### Table 1–S2. Results of linear models

|  | **Herb layer cover [%]** | | | **Herb layer height [log(cm)+1]** | | | **Cover of grasses [log(%)+1]** | | | **Moss layer cover [log(%)+1]** | | | **Moss layer height [mm]** | | |
| --- | --- | --- | --- | --- | --- | --- | --- | --- | --- | --- | --- | --- | --- | --- | --- |
| *Predictors* | *Estimates* | *CI* | *p* | *Estimates* | *CI* | *p* | *Estimates* | *CI* | *p* | *Estimates* | *CI* | *p* | *Estimates* | *CI* | *p* |
| (Intercept) | 74.82 | 64.12 – 85.52 | **<0.001** | 3.19 | 2.97 – 3.42 | **<0.001** | 4.19 | 3.85 – 4.54 | **<0.001** | 1.87 | 0.83 – 2.91 | **0.001** | 13.77 | 5.59 – 21.95 | **0.001** |
| Elevation | 0.01 | -0.00 – 0.02 | 0.053 | -0.00029 | -0.00 – -0.00 | **0.021** | -0.00 | -0.00 – 0.00 | 0.127 | 0.00 | -0.00 – 0.00 | 0.111 | -0.00 | -0.01 – 0.01 | 0.809 |
| Management Time [intermediate] | -0.65 | -10.36 – 9.06 | 0.893 | 0.01 | -0.19 – 0.22 | 0.884 | -0.17 | -0.49 – 0.14 | 0.272 | 0.23 | -0.72 – 1.17 | 0.632 | 1.18 | -6.25 – 8.61 | 0.751 |
| Management Time  [late] | 3.85 | -6.82 – 14.52 | 0.472 | 0.04 | -0.19 – 0.26 | 0.746 | -0.14 | -0.49 – 0.20 | 0.415 | 0.49 | -0.55 – 1.53 | 0.347 | 1.27 | -6.90 – 9.43 | 0.756 |
| Management Type  [mowing] | -4.83 | -11.75 – 2.08 | 0.166 | -0.03 | -0.18 – 0.11 | 0.643 | -0.07 | -0.29 – 0.16 | 0.561 | 0.19 | -0.49 – 0.86 | 0.582 | -0.69 | -5.98 – 4.59 | 0.793 |
| Observations | 52 | | | 52 | | | 52 | | | 52 | | | 52 | | |
| F (df), *p* | 2.042 (4,47), 0.1037 | | | 1.556 (4,47), 0.2017 | | | 1.465 (4,47), 0.2279 | | | 1.838 (4,47), 0.1372 | | | 0.04337 (4,47), 0.9963 | | |
| R^2^ / R^2^adjusted | 0.148 / 0.076 | | | 0.117 / 0.042 | | | 0.111 / 0.035 | | | 0.135 / 0.062 | | | 0.004 / -0.081 | | |

### Table 1–S2. Results of linear models (Continued)

|  | **Litter layer cover [log(%)+1]** | | | **Litter layer height [log(cm)+1]** | | | **Bare soil [log(%)+1]** | | | **Top soil pH (H2O)** | | | **Top soil pH (KCl)** | | |
| --- | --- | --- | --- | --- | --- | --- | --- | --- | --- | --- | --- | --- | --- | --- | --- |
| *Predictors* | *Estimates* | *CI* | *p* | *Estimates* | *CI* | *p* | *Estimates* | *CI* | *p* | *Estimates* | *CI* | *p* | *Estimates* | *CI* | *p* |
| (Intercept) | 2.94 | 2.40 – 3.48 | **<0.001** | 3.23 | 2.45 – 4.00 | **<0.001** | 1.41 | 0.43 – 2.39 | **0.006** | 4.78 | 4.49 – 5.07 | **<0.001** | 3.80 | 3.57 – 4.02 | **<0.001** |
| Elevation | -0.00 | -0.00 – 0.00 | 0.192 | 0.00 | -0.00 – 0.00 | 0.387 | -0.00 | -0.00 – 0.00 | 0.576 | -0.00 | -0.00 – 0.00 | 0.939 | 0.00 | -0.00 – 0.00 | 0.258 |
| Management Time [intermediate] | 0.07 | -0.27 – 0.42 | 0.674 | -0.22 | -0.72 – 0.28 | 0.379 | -0.10 | -0.73 – 0.54 | 0.762 | 0.07 | -0.12 – 0.26 | 0.463 | 0.05 | -0.10 – 0.20 | 0.489 |
| Management Time  [late] | -0.33 | -0.82 – 0.16 | 0.180 | -0.42 | -1.13 – 0.28 | 0.235 | -0.69 | -1.57 – 0.20 | 0.126 | 0.09 | -0.18 – 0.35 | 0.509 | -0.00 | -0.21 – 0.20 | 0.982 |
| Management Type  [mowing] | -0.42 | -0.95 – 0.12 | 0.123 | -0.18 | -0.95 – 0.60 | 0.649 | -0.38 | -1.35 – 0.60 | 0.438 | 0.00 | -0.28 – 0.29 | 0.975 | -0.01 | -0.24 – 0.21 | 0.923 |
| Observations | 52 | | | 52 | | | 52 | | | 52 | | | 52 | | |
| F (df), *p* | 1.399 (4,47), 0.2489 | | | 1.085 (4,47), 0.3748 | | | 1.158 (4,47), 0.3415 | | | 0.5249 (4,47), 0.7179 | | | 0.5379 (4,47), 0.7086 | | |
| R^2^ / R^2^adjusted | 0.106 / 0.030 | | | 0.085 / 0.007 | | | 0.090 / 0.012 | | | 0.043 / -0.039 | | | 0.044 / -0.038 | | |

# **Supporting information S3**: Trait - environment models with summer aridity and management factors (Performance: (generalised) linear mixed effects models / CV: linear models).

### Table 1-S3. Results for performance of vegetative traits

|  | **Vegetative Height [log(mm)+1]** | | | **Leaf Number [n]** | | | **Leaf Area [mm^2^]** | | |
| --- | --- | --- | --- | --- | --- | --- | --- | --- | --- |
| *Predictors* | *Estimates* | *CI* | *p* | *Estimates* | *CI* | *p* | *Estimates* | *CI* | *p* |
| (Intercept) | 4.53 | 4.33 – 4.73 | **<0.001** | 4.68 | 3.88 – 5.49 | **<0.001** | 2974.51 | 2226.13 – 3722.89 | **<0.001** |
| Summer aridity (linear, polynomial 1) | 0.11 | 0.05 – 0.16 | **0.001** | -6.98 | -12.50 – -1.46 | **0.017** | 626.54 | 418.67 – 834.41 | **<0.001** |
| Summer aridity (polynomial 2) |  |  |  | 1.56 | -5.26 – 8.38 | 0.656 |  |  |  |
| Summer aridity (polynomial 2) |  |  |  | 8.75 | 2.81 – 14.70 | **0.006** |  |  |  |
| Management type: mowing | -0.12 | -0.28 – 0.03 | 0.130 | 0.00 | -0.61 – 0.62 | 0.999 | -309.80 | -897.14 – 277.54 | 0.307 |
| Management time: intermediate | -0.11 | -0.33 – 0.11 | 0.350 | 0.24 | -0.64 – 1.11 | 0.599 | -280.21 | -1105.91 – 545.50 | 0.509 |
| Management time: late | 0.17 | -0.07 – 0.42 | 0.179 | 0.45 | -0.55 – 1.45 | 0.382 | 615.00 | -300.14 – 1530.14 | 0.194 |
| **Random Effects** | | | | | | | | | |
| σ^2^ | 0.06 | | | 1.11 | | | 630942.41 | | |
| τ_00_ | 0.03 _IDpopulation_ | | | 0.47 _IDpopulation_ | | | 420478.66 _IDpopulation_ | | |
| ICC | 0.36 | | | 0.30 | | | 0.40 | | |
| N | 52 _IDpopulation_ | | | 52 _IDpopulation_ | | | 52 _IDpopulation_ | | |
| Observations | 624 | | | 624 | | | 312 | | |
| Marginal R^2^ / Conditional R^2^ | 0.250 / 0.518 | | | 0.128 / 0.386 | | | 0.378 / 0.627 | | |

Note: Summer aridity was mean centered and scaled.

### Table 2-S3. Results for performance of generative traits

|  | **Flower Stem Height [mm]** | | | **Inflorescence Number [*n*]** | | | **Inflorescence Diameter [mm]** | | |
| --- | --- | --- | --- | --- | --- | --- | --- | --- | --- |
| *Predictors* | *Estimates* | *CI* | *p* | *Incidence Rate Ratios* | *CI* | *p* | *Estimates* | *CI* | *p* |
| (Intercept) | 413.28 | 346.63 – 479.93 | **<0.001** | 1.02 | 0.49 – 2.13 | 0.962 | 14.89 | 12.84 – 16.94 | **<0.001** |
| Summer aridity | 58.59 | 41.68 – 75.51 | **<0.001** | 1.50 | 1.23 – 1.82 | **<0.001** | 1.37 | 0.84 – 1.89 | **<0.001** |
| Management type: mowing | -32.26 | -81.61 – 17.09 | 0.206 | 1.40 | 0.81 – 2.44 | 0.233 | -0.44 | -1.97 – 1.09 | 0.574 |
| Management time: intermediate | -18.94 | -90.31 – 52.44 | 0.606 | 1.57 | 0.70 – 3.51 | 0.270 | -0.54 | -2.74 – 1.65 | 0.631 |
| Management time: late | 18.88 | -57.86 – 95.61 | 0.632 | 1.99 | 0.83 – 4.78 | 0.124 | 0.26 | -2.10 – 2.62 | 0.828 |
| **Random Effects** | | | | | | | | | |
| σ^2^ | 4521.53 | | | 0.37 | | | 5.61 | | |
| τ_00_ | 2630.74 _IDpopulation_ | | | 0.40 _IDpopulation_ | | | 2.34 _IDpopulation_ | | |
| ICC | 0.37 | | | 0.52 | | | 0.29 | | |
| N | 50 _IDpopulation_ | | | 52 _IDpopulation_ | | | 50 _IDpopulation_ | | |
| Observations | 506 | | | 624 | | | 506 | | |
| Marginal R^2^ / Conditional R^2^ | 0.322 / 0.571 | | | 0.288 / 0.656 | | | 0.188 / 0.427 | | |

Note: Summer aridity was mean centered and scaled.

### Table 3-S3. Results for performance of physiological traits

|  | **Leaf Dry Biomass [log(mg)+1]** | | | **Specific Leaf Area [mm^2^ mg^-1^]** | | | **Leaf Dry Matter Content [mg g^-1^]** | | |
| --- | --- | --- | --- | --- | --- | --- | --- | --- | --- |
| *Predictors* | *Estimates* | *CI* | *p* | *Estimates* | *CI* | *p* | *Estimates* | *CI* | *p* |
| (Intercept) | 4.85 | 4.56 – 5.14 | **<0.001** | 21.27 | 18.60 – 23.95 | **<0.001** | 136.48 | 121.40 – 151.56 | **<0.001** |
| Summer aridity | 0.22 | 0.14 – 0.31 | **<0.001** | 0.27 | -0.47 – 1.02 | 0.474 | -3.74 | -7.93 – 0.44 | 0.086 |
| Management type: mowing | 0.03 | -0.20 – 0.25 | 0.810 | -3.05 | -5.15 – -0.95 | **0.006** | 10.27 | -1.56 – 22.11 | 0.096 |
| Management time: intermediate | -0.01 | -0.32 – 0.31 | 0.967 | -0.89 | -3.83 – 2.06 | 0.559 | -0.66 | -17.30 – 15.98 | 0.939 |
| Management time: late | 0.32 | -0.03 – 0.68 | 0.080 | -1.80 | -5.07 – 1.47 | 0.285 | -1.45 | -19.89 – 16.99 | 0.878 |
| **Random Effects** | | | | | | | | | |
| σ^2^ | 0.10 | | | 8.21 | | | 424.44 | | |
| τ_00_ | 0.06 _IDpopulation_ | | | 5.34 _IDpopulation_ | | | 142.67 _IDpopulation_ | | |
| ICC | 0.38 | | | 0.39 | | | 0.25 | | |
| N | 52 _IDpopulation_ | | | 52 _IDpopulation_ | | | 52 _IDpopulation_ | | |
| Observations | 312 | | | 312 | | | 312 | | |
| Marginal R^2^ / Conditional R^2^ | 0.376 / 0.612 | | | 0.145 / 0.482 | | | 0.040 / 0.282 | | |

Note: Summer aridity was mean centered and scaled.

### Table 4-S3. Results for variability of vegetative traits

|  | **CV Vegetative Height** | | | **CV Leaf Number** | | | **CV Leaf Area** | | |
| --- | --- | --- | --- | --- | --- | --- | --- | --- | --- |
| *Predictors* | *Estimates* | *CI* | *p* | *Estimates* | *CI* | *p* | *Estimates* | *CI* | *p* |
| (Intercept) | 0.05 | 0.02 – 0.07 | **<0.001** | 0.27 | 0.22 – 0.31 | **<0.001** | 0.21 | 0.09 – 0.33 | **0.002** |
| Summer aridity (linear, polynomial 1) | -0.05 | -0.10 – -0.01 | **0.021** | -0.02 | -0.03 – -0.01 | **0.005** | -0.07 | -0.10 – -0.03 | **<0.001** |
| Summer aridity (polynomial 2) | -0.06 | -0.11 – -0.01 | **0.037** |  |  |  |  |  |  |
| Summer aridity (polynomial 3) | -0.06 | -0.11 – -0.02 | **0.011** |  |  |  |  |  |  |
| Management type: mowing | 0.00 | -0.01 – 0.02 | 0.637 | -0.02 | -0.06 – 0.01 | 0.186 | 0.03 | -0.06 – 0.13 | 0.525 |
| Management time: intermediate | 0.00 | -0.02 – 0.02 | 0.930 | -0.05 | -0.10 – -0.00 | 0.054 | 0.02 | -0.12 – 0.15 | 0.805 |
| Management time: late | 0.00 | -0.02 – 0.03 | 0.826 | -0.06 | -0.12 – -0.01 | **0.031** | 0.06 | -0.08 – 0.21 | 0.398 |
| Observations | 52 | | | 52 | | | 52 | | |
| F (df), *p* | 3.453 (6,45), 0.007 | | | 7.546 (4,47), <0.001 | | | 3.82 (4,47), 0.009 | | |
| R^2^ / adjusted R^2^ | 0.315 / 0.224 | | | 0.391 / 0.339 | | | 0.245 / 0.181 | | |

Note: Summer aridity was mean centered and scaled.

### Table 5-S3. Results for variability of generative traits

|  | **CV Flower Stem Height** | | | **CV Inflorescence Number** | | | **CV Inflorescence Diameter** | | |
| --- | --- | --- | --- | --- | --- | --- | --- | --- | --- |
| *Predictors* | *Estimates* | *CI* | *p* | *Estimates* | *CI* | *p* | *Estimates* | *CI* | *p* |
| (Intercept) | 0.17 | 0.05 – 0.30 | **0.011** | 0.54 | -0.25 – 1.34 | 0.185 | 0.17 | 0.12 – 0.23 | **<0.001** |
| Summer aridity | -0.04 | -0.08 – -0.01 | **0.015** | -0.37 | -0.56 – -0.17 | **0.001** | -0.02 | -0.03 – -0.00 | **0.038** |
| Management type: mowing | -0.04 | -0.14 – 0.05 | 0.360 | -0.07 | -0.64 – 0.51 | 0.819 | 0.03 | -0.02 – 0.07 | 0.243 |
| Management time: intermediate | 0.05 | -0.09 – 0.19 | 0.472 | 0.46 | -0.38 – 1.31 | 0.288 | -0.05 | -0.11 – 0.02 | 0.157 |
| Management time: late | 0.04 | -0.10 – 0.19 | 0.571 | 0.10 | -0.82 – 1.01 | 0.834 | -0.03 | -0.10 – 0.03 | 0.353 |
| Observations | 48 | | | 50 | | | 48 | | |
| F (df), *p* | 2.102 (5,46), 0.097 | | | 5.073 (4,45), 0.002 | | | 2.237 (4,43), 0.08 | | |
| R^2^ / adjusted R^2^ | 0.164 / 0.086 | | | 0.311 / 0.250 | | | 0.172 / 0.095 | | |

Note: Summer aridity was mean centered and scaled.

### Table 6-S3. Results for variability of physiological traits

|  | **CV Leaf Dry Biomass** | | | **CV Specific Leaf Area** | | | **CV Leaf Dry Matter Content** | | |
| --- | --- | --- | --- | --- | --- | --- | --- | --- | --- |
| *Predictors* | *Estimates* | *CI* | *p* | *Estimates* | *CI* | *p* | *Estimates* | *CI* | *p* |
| (Intercept) | 0.19 | 0.09 – 0.30 | **0.001** | 0.08 | -0.03 – 0.19 | 0.158 | 0.11 | 0.07 – 0.16 | **<0.001** |
| Summer aridity | -0.05 | -0.08 – -0.02 | **0.004** | -0.04 | -0.07 – -0.01 | **0.014** | -0.01 | -0.03 – 0.00 | 0.073 |
| Management type: mowing | 0.04 | -0.05 – 0.12 | 0.414 | -0.01 | -0.10 – 0.08 | 0.859 | -0.01 | -0.05 – 0.03 | 0.628 |
| Management time: intermediate | 0.08 | -0.04 – 0.20 | 0.187 | 0.06 | -0.06 – 0.18 | 0.353 | 0.03 | -0.03 – 0.08 | 0.319 |
| Management time: late | 0.08 | -0.05 – 0.21 | 0.237 | 0.10 | -0.03 – 0.24 | 0.142 | 0.03 | -0.03 – 0.09 | 0.319 |
| Observations | 52 | | | 52 | | | 52 | | |
| F (df), *p* | 2.753 (4,47), 0.039 | | | 1.956 (4,47), 0.117 | | | 1.01 (4,47), 0.412 | | |
| R^2^ / adjusted R^2^ | 0.190 / 0.121 | | | 0.143 / 0.070 | | | 0.079 / 0.001 | | |

Note: Summer aridity was mean centered and scaled.

# **Supporting information S4**: Trait – environment models with elevation and management factors (Performance: (generalised) linear mixed effects models / CV: linear models).

### Table 1-S4. Results for performance of vegetative traits

|  | **Vegetative Height [log(mm)+1]** | | | **Leaf Number [n]** | | | **Leaf Area [mm^2^]** | | |
| --- | --- | --- | --- | --- | --- | --- | --- | --- | --- |
| *Predictors* | *Estimates* | *CI* | *p* | *Estimates* | *CI* | *p* | *Estimates* | *CI* | *p* |
| (Intercept) | 4.52 | 4.33 – 4.71 | **<0.001** | 4.91 | 4.20 – 5.61 | **<0.001** | 2941.92 | 2290.67 – 3593.17 | **<0.001** |
| Elevation (linear, polynomial 1) | 0.11 | 0.06 – 0.17 | **<0.001** | -8.46 | -13.09 – -3.82 | **0.001** | 699.22 | 520.88 – 877.56 | **<0.001** |
| Elevation (polynomial 2) |  |  |  | 9.25 | 2.91 – 15.59 | **0.006** |  |  |  |
| Elevation (polynomial 3) |  |  |  | 5.47 | 0.84 – 10.10 | **0.025** |  |  |  |
| Management type: mowing | -0.12 | -0.27 – 0.03 | 0.136 | -0.11 | -0.65 – 0.43 | 0.696 | -276.04 | -791.79 – 239.71 | 0.300 |
| Management time: intermediate | -0.11 | -0.32 – 0.10 | 0.319 | 0.19 | -0.57 – 0.95 | 0.622 | -316.77 | -1041.36 – 407.83 | 0.396 |
| Management time: late | 0.18 | -0.05 – 0.42 | 0.131 | 0.18 | -0.70 – 1.06 | 0.692 | 670.80 | -125.22 – 1466.81 | 0.105 |
| **Random Effects** | | | | | | | | | |
| σ^2^ | 0.06 | | | 1.11 | | | 630942.42 | | |
| τ_00_ | 0.03 _IDpopulation_ | | | 0.34 _IDpopulation_ | | | 300771.43 _IDpopulation_ | | |
| ICC | 0.34 | | | 0.23 | | | 0.32 | | |
| N | 52 _IDpopulation_ | | | 52 _IDpopulation_ | | | 52 _IDpopulation_ | | |
| Observations | 624 | | | 624 | | | 312 | | |
| Marginal R^2^ / Conditional R^2^ | 0.268 / 0.517 | | | 0.166 / 0.360 | | | 0.445 / 0.624 | | |

Note: Elevation was mean centered and scaled.

### Table 2-S4. Results for performance of generative traits

|  | **Flower Stem Height [mm]** | | | **Inflorescence Number [*n*]** | | | **Inflorescence Diameter [mm]** | | |
| --- | --- | --- | --- | --- | --- | --- | --- | --- | --- |
| *Predictors* | *Estimates* | *CI* | *p* | *Incidence Rate Ratios* | *CI* | *p* | *Estimates* | *CI* | *p* |
| (Intercept) | 400.97 | 337.74 – 464.21 | **<0.001** | 0.95 | 0.46 – 1.96 | 0.892 | 14.64 | 12.77 – 16.50 | **<0.001** |
| Elevation | 62.36 | 46.50 – 78.22 | **<0.001** | 1.49 | 1.24 – 1.80 | **<0.001** | 1.51 | 1.04 – 1.98 | **<0.001** |
| Management type: mowing | -27.14 | -74.47 – 20.19 | 0.267 | 1.44 | 0.84 – 2.49 | 0.189 | -0.37 | -1.77 – 1.03 | 0.609 |
| Management time: intermediate | -14.18 | -82.33 – 53.96 | 0.685 | 1.61 | 0.73 – 3.55 | 0.240 | -0.44 | -2.44 – 1.57 | 0.674 |
| Management time: late | 31.13 | -41.62 – 103.89 | 0.407 | 2.16 | 0.92 – 5.10 | 0.078 | 0.55 | -1.60 – 2.69 | 0.620 |
| **Random Effects** | | | | | | | | | |
| σ^2^ | 4521.61 | | | 0.37 | | | 5.62 | | |
| τ_00_ | 2374.64 _IDpopulation_ | | | 0.38 _IDpopulation_ | | | 1.87 _IDpopulation_ | | |
| ICC | 0.34 | | | 0.51 | | | 0.25 | | |
| N | 50 _IDpopulation_ | | | 52 _IDpopulation_ | | | 50 _IDpopulation_ | | |
| Observations | 506 | | | 624 | | | 506 | | |
| Marginal R^2^ / Conditional R^2^ | 0.358 / 0.579 | | | 0.295 / 0.654 | | | 0.228 / 0.421 | | |

Note: Elevation was mean centered and scaled.

### Table 3-S4. Results for performance of physiological traits

|  | **Leaf Dry Biomass [log(mg)+1]** | | | **Specific Leaf Area [mm^2^ mg^-1^]** | | | **Leaf Dry Matter Content [mg g^-1^]** | | |
| --- | --- | --- | --- | --- | --- | --- | --- | --- | --- |
| *Predictors* | *Estimates* | *CI* | *p* | *Estimates* | *CI* | *p* | *Estimates* | *CI* | *p* |
| (Intercept) | 4.83 | 4.57 – 5.10 | **<0.001** | 21.38 | 18.76 – 24.00 | **<0.001** | 136.06 | 121.56 – 150.56 | **<0.001** |
| Elevation | 0.24 | 0.17 – 0.32 | **<0.001** | 0.46 | -0.25 – 1.18 | 0.211 | -5.00 | -8.97 – -1.03 | **0.017** |
| Management type: mowing | 0.04 | -0.17 – 0.25 | 0.705 | -3.05 | -5.12 – -0.97 | **0.006** | 10.12 | -1.36 – 21.61 | 0.091 |
| Management time: intermediate | -0.01 | -0.31 – 0.28 | 0.921 | -1.01 | -3.92 – 1.91 | 0.502 | 0.10 | -16.03 – 16.24 | 0.990 |
| Management time: late | 0.35 | 0.03 – 0.67 | **0.039** | -1.91 | -5.11 – 1.29 | 0.247 | -1.08 | -18.80 – 16.64 | 0.905 |
| **Random Effects** | | | | | | | | | |
| σ^2^ | 0.10 | | | 8.21 | | | 424.44 | | |
| τ_00_ | 0.05 _IDpopulation_ | | | 5.19 _IDpopulation_ | | | 130.51 _IDpopulation_ | | |
| ICC | 0.33 | | | 0.39 | | | 0.24 | | |
| N | 52 _IDpopulation_ | | | 52 _IDpopulation_ | | | 52 _IDpopulation_ | | |
| Observations | 312 | | | 312 | | | 312 | | |
| Marginal R^2^ / Conditional R^2^ | 0.418 / 0.610 | | | 0.154 / 0.481 | | | 0.059 / 0.280 | | |

Note: Elevation was mean centered and scaled.

### **Figure 1-S4.** Changes in the plant functional trait performance of *Arnica montana* with increasing elevation (high scores). Solid lines indicate significant model trends (*p* < 0.05) and dashed lines non-significant trends with the grey-shaded 95% confidence interval of the model. Figure parts A to C show trends for vegetative traits, D to F for generative traits and G to I for physiological traits. See Table 1 to 3 in Supp. Info. S4 for detailed model results.

**
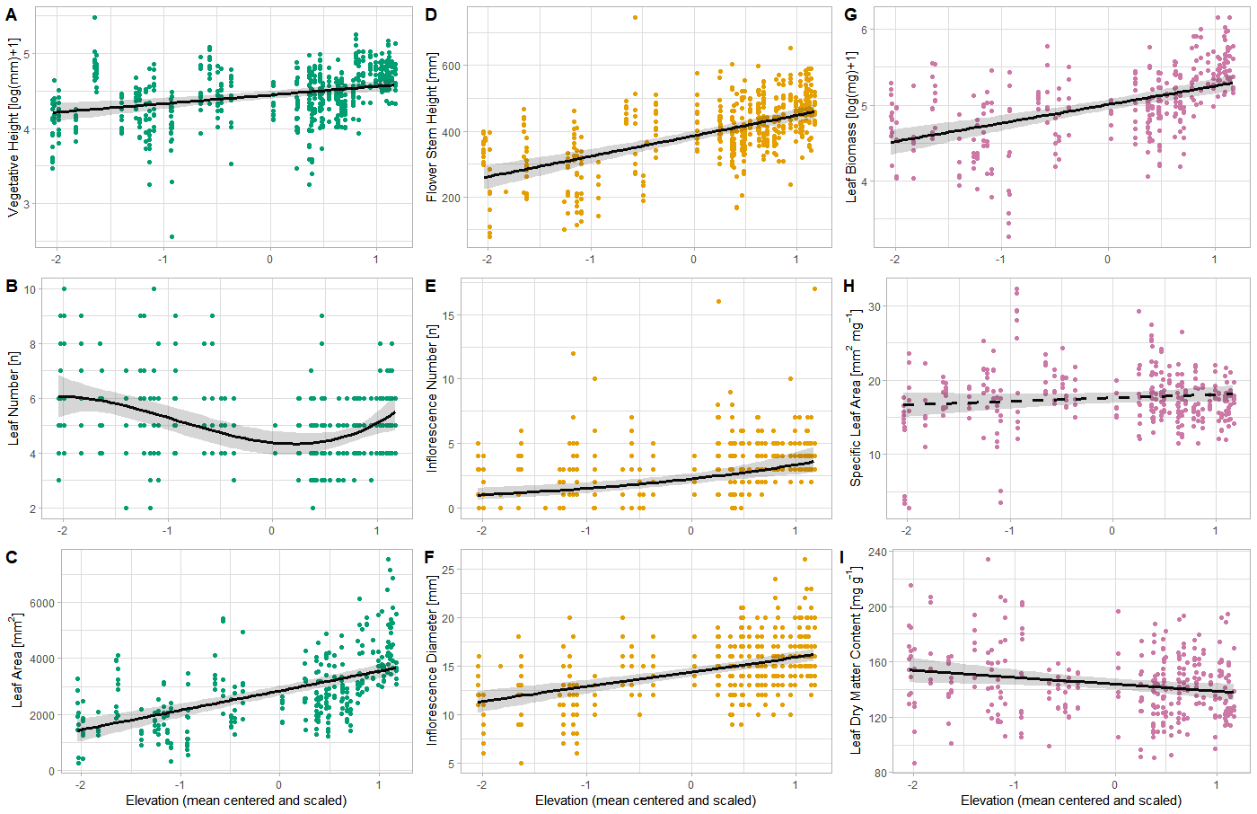
**

### Table 4-S4. Results for variability of vegetative traits

|  | **CV Vegetative Height** | | | **CV Leaf Number** | | | **CV Leaf Area** | | |
| --- | --- | --- | --- | --- | --- | --- | --- | --- | --- |
| *Predictors* | *Estimates* | *CI* | *p* | *Estimates* | *CI* | *p* | *Estimates* | *CI* | *p* |
| (Intercept) | 0.04 | 0.02 – 0.07 | **<0.001** | 0.27 | 0.23 – 0.32 | **<0.001** | 0.21 | 0.10 – 0.33 | **0.001** |
| Elevation (linear, polynomial 1) | -0.05 | -0.09 – -0.00 | **0.043** | -0.02 | -0.03 – -0.01 | **0.005** | -0.07 | -0.10 – -0.04 | **<0.001** |
| Elevation (polynomial 2) | -0.06 | -0.12 – -0.00 | **0.048** |  |  |  |  |  |  |
| Management type: mowing | 0.00 | -0.01 – 0.02 | 0.704 | -0.03 | -0.06 – 0.01 | 0.165 | 0.03 | -0.06 – 0.12 | 0.557 |
| Management time: intermediate | 0.01 | -0.02 – 0.03 | 0.574 | -0.05 | -0.10 – -0.00 | 0.051 | 0.02 | -0.11 – 0.15 | 0.762 |
| Management time: late | 0.00 | -0.03 – 0.03 | 0.889 | -0.07 | -0.12 – -0.01 | **0.021** | 0.06 | -0.08 – 0.20 | 0.426 |
| Observations | 52 | | | 52 | | | 52 | | |
| F (df), *p* | 3.033 (5,46), 0.019 | | | 7.621 (4,47), <0.001 | | | 5.169 (4,47), 0.002 | | |
| R^2^ / adjusted R^2^ | 0.248 / 0.166 | | | 0.393 / 0.342 | | | 0.306 / 0.246 | | |

Note: Elevation was mean centered and scaled.

### Table 5-S4. Results for variability of generative traits

|  | **CV Flower Stem Height** | | | **CV Inflorescence Number** | | | **CV Inflorescence Diameter** | | |
| --- | --- | --- | --- | --- | --- | --- | --- | --- | --- |
| *Predictors* | *Estimates* | *CI* | *p* | *Estimates* | *CI* | *p* | *Estimates* | *CI* | *p* |
| (Intercept) | 0.18 | 0.06 – 0.30 | **0.006** | 0.63 | -0.15 – 1.42 | 0.122 | 0.18 | 0.12 – 0.23 | **<0.001** |
| Elevation | -0.05 | -0.08 – -0.02 | **0.002** | -0.36 | -0.55 – -0.16 | **0.001** | -0.02 | -0.03 – -0.00 | **0.014** |
| Management type: mowing | -0.05 | -0.14 – 0.04 | 0.294 | -0.09 | -0.67 – 0.48 | 0.753 | 0.02 | -0.02 – 0.06 | 0.266 |
| Management time: intermediate | 0.05 | -0.08 – 0.18 | 0.457 | 0.42 | -0.42 – 1.27 | 0.332 | -0.05 | -0.11 – 0.01 | 0.140 |
| Management time: late | 0.04 | -0.10 – 0.17 | 0.602 | -0.01 | -0.91 – 0.90 | 0.991 | -0.03 | -0.10 – 0.03 | 0.299 |
| Observations | 48 | | | 50 | | | 48 | | |
| F (df), *p* | 3.171 (4,43), 0.023 | | | 5.051 (4,45), 0.002 | | | 2.77 (4,43), 0.039 | | |
| R^2^ / adjusted R^2^ | 0.228 / 0.156 | | | 0.310 / 0.249 | | | 0.205 / 0.131 | | |

Note: Elevation was mean centered and scaled.

### Table 6-S4. Results for variability of generative traits

|  | **CV Leaf Dry Biomass** | | | **CV Specific Leaf Area** | | | **CV Leaf Dry Matter Content** | | |
| --- | --- | --- | --- | --- | --- | --- | --- | --- | --- |
| *Predictors* | *Estimates* | *CI* | *p* | *Estimates* | *CI* | *p* | *Estimates* | *CI* | *p* |
| (Intercept) | 0.20 | 0.09 – 0.30 | **0.001** | 0.08 | -0.03 – 0.19 | 0.144 | 0.11 | 0.07 – 0.16 | **<0.001** |
| Elevation | -0.05 | -0.08 – -0.02 | **0.001** | -0.05 | -0.08 – -0.02 | **0.001** | -0.02 | -0.03 – -0.01 | **0.008** |
| Management type: mowing | 0.03 | -0.05 – 0.12 | 0.440 | -0.01 | -0.09 – 0.07 | 0.819 | -0.01 | -0.05 – -0.03 | 0.597 |
| Management time: intermediate | 0.08 | -0.03 – 0.20 | 0.172 | 0.06 | -0.05 – 0.18 | 0.290 | 0.03 | -0.02 – 0.07 | 0.296 |
| Management time: late | 0.08 | -0.05 – 0.20 | 0.260 | 0.10 | -0.03 – 0.23 | 0.121 | 0.03 | -0.02 – 0.08 | 0.321 |
| Observations | 52 | | | 52 | | | 52 | | |
| F (df), *p* | 3.339 (4,47), 0.017 | | | 3.185 (4,47), 0.021 | | | 2.06 (4,47), 0.101 | | |
| R^2^ / adjusted R^2^ | 0.221 / 0.155 | | | 0.213 / 0.146 | | | 0.144 / 0.091 | | |

Note: Elevation was mean centered and scaled.

### **Figure 2-S4.** Changes in the variability (CV, Coefficient of Variation) of plant functional trait of *Arnica montana* with increasing elevation (high scores). Solid lines indicate significant model trends (*p* < 0.05) with the grey-shaded 95% confidence interval of the model. Figure parts A to C show trends for vegetative traits, D to F: for generative traits and G to I: for physiological traits. See Table 4 to 6 in Supp. Info. S4 for detailed model results.


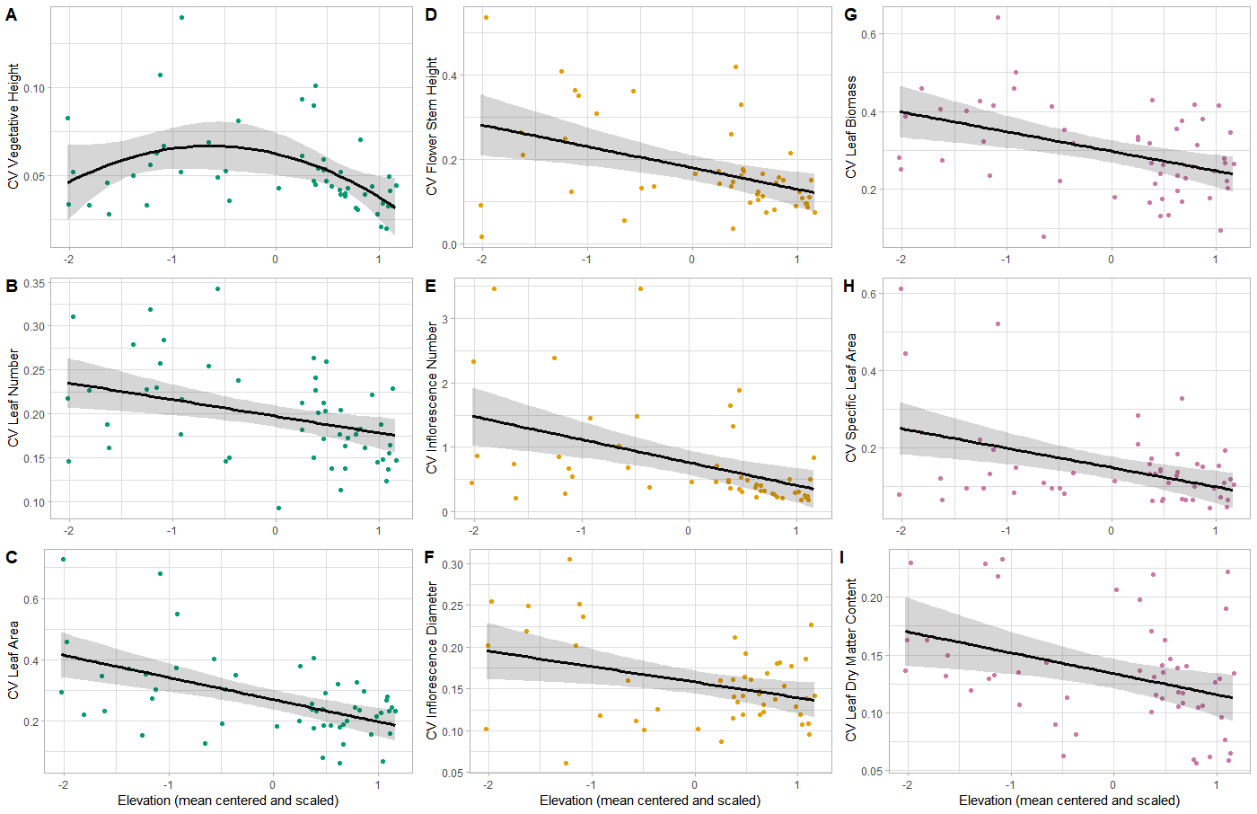


References

DWD Climate Data Center (2019a). Multi-annual grids of precipitation height over Germany 1981-2010, version v1.0. Retrieved from https://opendata.dwd.de/climate_environment/CDC/grids_germany/multi_annual/

DWD Climate Data Center (2019b). Multi-annual means of grids of air temperature (2m) over Germany 1981-2010, version v1.0. Retrieved from https://opendata.dwd.de/climate_environment/CDC/grids_germany/multi_annual/
